# Relationship between paramacular thinning, cerebral vasculopathy, and hematological risk factors in sickle cell disease

**DOI:** 10.3389/fmed.2023.1226210

**Published:** 2023-08-28

**Authors:** Christophe Orssaud, Edouard Flamarion, Adrien Michon, Brigitte Ranque, Jean Benoit Arlet

**Affiliations:** ^1^Functional Unity of Ophthalmology, ERN EYE, Ophthalmological Rare Diseases Center, Georges Pompidou European Hospital, Assistance Publique-Hopitaux de Paris, Paris, France; ^2^Internal Medicine Department, French National Sickle Cell Referral Center, European Hospital Georges Pompidou, Paris, France; ^3^Faculty of Medicine Université Paris Cité, Paris, France; ^4^Université Paris Cité, INSERM UMR-S970, Paris, France; ^5^Laboratoire d'Excellence sur le globule rouge GR-ex, Paris, France; ^6^INSERM U, CNRS 8254, Institut IMAGINE, Hôpital Necker, Assistance Publique-Hôpitaux de Paris, Paris, France

**Keywords:** cerebral vasculopathy, hemolysis (red blood cells), maculopathy, OCT, optical coherence tomography angiography (OCT-A), sickle cell disease

## Abstract

**Purpose:**

To identify risk factors for sickle cell maculopathy due to hematological parameters (especially anemia and hemolysis) or cerebral vasculopathy.

**Methods:**

This retrospective study was conducted at a Referral Center. The follow-up included optical coherent tomography**/**optical coherent tomography angiography, neuro-radiological imaging, and a hematological assessment **(**hemoglobin, hemoglobin S level, reticulocytes, mean corpuscular volume, bilirubin, and lactate dehydrogenase).

**Results:**

Hundred and thirty-two sickle cell patients were included. Maculopathy was observed in 127 eyes of SS patients and 10 eyes of SC patients (*p* < 0.001), unrelated to peripheral retinopathy. Cerebral vasculopathy was more frequent in SS patients (*p* < 0.001) and was also associated with the presence of maculopathy (*p* = 0.049), and it was related to peripheral retinopathy (*p* < 0.001). All biological parameters significantly differed according to the genotype (*p* < 0.001) but not according to the presence of cerebral vasculopathy or maculopathy. In the multivariate analysis, reticulocytes and bilirubin were associated with the presence of cerebral vasculopathy and maculopathy.

**Conclusion:**

The data obtained were consistent with the role of anemia or hemolysis markers in cerebral vasculopathy and macular involvement. As a trend of hemolysis appears to be a risk factor for these complications, this validates the use of preventive plasmapheresis in these patients.

## Introduction

Sickle cell disease (SCD) is a group of autosomal recessive hemoglobinopathies. It is characterized by the presence of an abnormal globin protein chain, hemoglobin S (HbS), resulting from a single amino acid substitution in the hemoglobin gene (1). The three main genotypes of SCD are the homozygous SS, double heterozygous SC, and sickle cell thalassemia HbS/thal. This abnormal HbS leads to rigid and fragile erythrocytes that may sickle under hypoxic, acidic, or dehydrated conditions, leading to acute vascular occlusions and chronic hemolytic anemia (1).

Retinal complications have been frequently reported in SCD, including salmon-patch hemorrhages, iridescent spots, black sunbursts in any area of the retina, and peripheral sickle retinopathy (PSR), predominantly observed in patients with the SC genotype or SCM (2, 3). In addition, since optical coherence tomography (OCT) and OCT angiography (OCT-A) are usually performed, involvement of the temporal paramacular retina is classically reported. This sickle cell maculopathy (SCM) is characterized by areas of retinal thinning associated with a reduction of vascular flow in both superficial capillary plexuses (SCPs) and deep capillary plexuses (DCPs), usually along the median temporal raphe (4–7). Besides this typical form, other atypical forms of SCM, as well as an enlargement of the foveal avascular zone (FAZ), has also been reported.

Its repartition according to the SS or SC genotypes is still debated, with contradictory data in the literature (8, 9). Several other questions regarding SCM are still unanswered. Its pathophysiological mechanisms and risk factors, as well as the age of the occurrence, are not clearly established. Its possible association with PSR is debated. It has been suggested that they could share the same mechanism as silent borderzone cerebral infarct (6). SCM would then be an ocular equivalent of cerebrovascular vasculopathy, especially in patients with the SS genotype (10). Other authors considered that macular involvement is due to localized arteriolar occlusions, leading first to transient and then to permanent capillary non-perfusion. Macular thinning would be the consequence of localized ischemia (11).

This study primarily aims to identify correlations between SCM, cerebral vasculopathy, and blood factors of hemolysis. We then discuss what these results can bring to the understanding of the risk factors and physiopathogeny of SCM.

## Patients and methods

### Patients

In this retrospective study, we analyzed the data of consecutive SCD patients referred with a routine ophthalmological assessment (at a steady state of their disease) in a single ophthalmological department from October 2018 to November 2019. These patients were followed-up in a national SCD referral center located in the same university hospital; they benefit from ophthalmological assessment each year. To study homogeneous population groups, we excluded heterozygous S-beta thalassemia patients. All the subjects were tested in accordance with the relevant guidelines and regulations. The last visit was considered if a patient was referred more than one time. The type of sickle hemoglobinopathy was determined by hemoglobin electrophoresis (HPLC) and obtained from the medical record. Patients were divided into two groups according to their genotype: SS patients and SC patients.

According to the national legislation, patients did not have to give consent, but they were informed that their data might be used anonymously and that they could oppose the usage. The local ethics committee (CERAPHP Center, Comité d'éthique de la recherche AP-HP Center) approved this retrospective study. It adhered to the tenets of the Declaration of Helsinki.

### Methods

Data on the past medical history of stroke or cerebral vasculopathy were retrospectively extracted from the electronic medical record charting system. These data were collected during routine follow-up outpatient visits using a standardized form that was regularly completed at each routine visit. Cerebral vasculopathy was defined as the presence of cerebral vessel stenosis, moyamoya disease, aneurysm, stroke, or silent cerebral infarct; therefore, it was only evaluable in patients who had undergone brain magnetic resonance imaging (MRI) or a brain computed tomography (CT) scan. Biological data suggestive of anemia, hemolysis, or blood hyperviscosity were collected from SS and SC patients: hemoglobin (Hb), HbS level, reticulocyte count, mean corpuscular volume (MCV), bilirubin, and the level of lactate dehydrogenase (LDH).

A complete routine screening ophthalmological examination was performed on the same day. It included far-sighted best-corrected visual acuity assessment using a visual acuity chart, the results of which were converted to the logarithm of the minimal angle of resolution (logMAR), slit-lamp examination of the anterior segment, dilated funduscopy, retinography, optical coherent tomography, and optical coherent tomography angiography (OCT-A). Pupil dilation of both eyes was performed using tropicamide 1%. Fluorescein angiography was performed only to confirm the presence of peripheral SCD retinopathy. The stage of this peripheral retinopathy was determined according to Goldberg's classification: stage 0: normal peripheral vascularization of the retina; stage I: peripheral arteriolar occlusion; stage II: peripheral arteriovenous anastomoses; stage III: preretinal neovascularization and fibrous proliferations (sea fan); stage IV: intravitreous hemorrhage; and stage V: tractional and/or rhegmatogenous retinal detachment.

### OCT analysis

Spectral-domain OCT images and OCT-A images of macular regions were obtained together using a Spectralis HRA–OCT2 (Heidelberg Engineering GmbH, Hamburg Germany). All OCT scans were performed in a dimly lit room after pupil dilation by a trained technician. Posterior pole volumes and angiographic images were recorded with 15° × 15° angle scans centered on the fovea with 61 raster lines, separated by 121 mm. OCT-A images were generated using the software, Spectralis HRA–OCT2. Automated segmentation of the different layers and the SCP and DCP was also achieved using the same software. The SCP was automatically localized according to the location of the inner limiting membrane, and the DCP was localized above the inner plexiform layer. Only one experienced investigator performed corrections when the segmentation was maladjusted. OCT scans were excluded when the quality was poor due to poor fixation during image acquisition and did not allow for a correct analysis of the retinal thickness and/or capillary plexus.

Automated retinal thickness color maps and measurements were obtained. The Early Treatment Diabetic Retinopathy Study (ETDRS) grid (composed of three concentric circles with diameters of 1, 3, and 6 mm, respectively), provided by Spectralis HRA–OCT2, was automatically positioned on the colored maps. The position of the grid was corrected if necessary. This allowed us to localize retinal thinning areas in the fovea central circle or in the different sectors (superior, temporal, nasal, and inferior) centered on the fovea.

The SD-OCT scans of each eye were performed and analyzed by only one examiner. Quantitative measurements of the retinal thickness were achieved on six different points. Four points were located at the junction of the concentric circles at 3 and 6 mm and on each of the two diagonal lines of the ETDRS grid for the upper temporal and lower temporal points. In addition, two points were located on the temporal horizontal meridian, at its junction with the two concentric circles at 3 and 6 mm of the ETDRS grid. Extrafoveal thinning was assessed qualitatively and quantitatively. The retinal extrafoveal thinning area was defined as an area of substantial reduction of the retinal layers.

SD-OCT scans were automatically aligned with OCT-A images of the SCP and DCP. It was then possible to verify whether a retinal thinning zone corresponded to an area of non-perfusion in the plexuses and whether areas of non-perfusion could be observed without any retinal atrophy.

Enlargement of the FAZ was qualitatively assessed on the OCT-A image. This enlargement was defined as circular enlarged avascular territories in the SCP or DCP, as well as a disruption of regular perifoveal vascular plexus beyond 1 mm diameter according to the ETDRS grid. Moreover, this diagnosis was retained in the presence of an abnormally flattened macular region. This flattening must concern the entire circumference and extend beyond the 1 mm diameter of the ETDRS grid.

**Figure 1:**
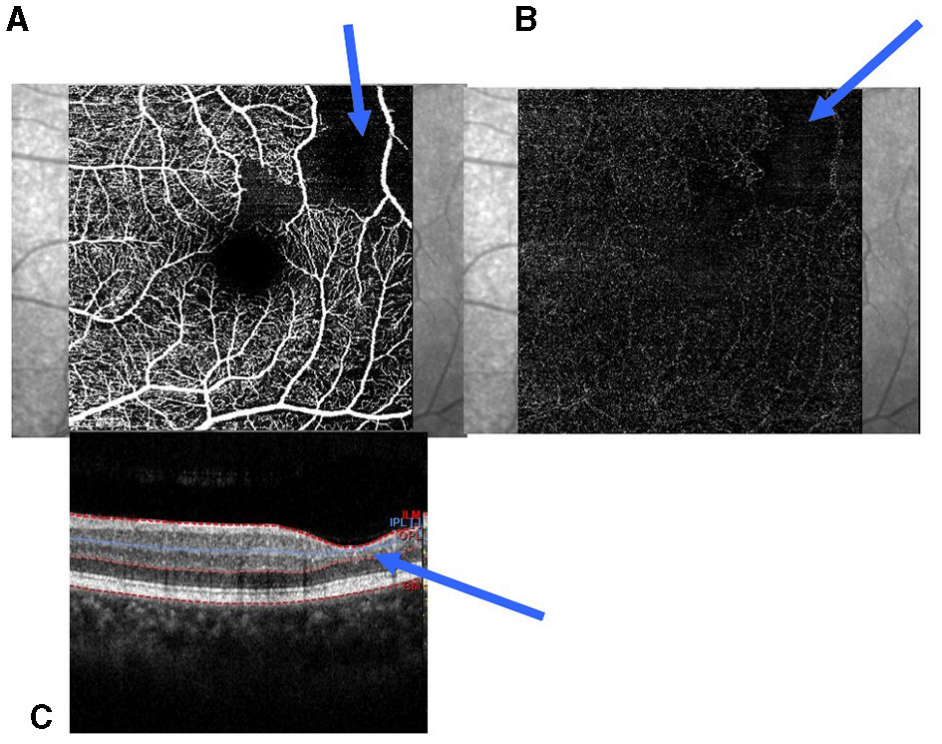
Parafoveolar retinal thinning and reduction of capillaries in the SCP and DCP. **(A)** Reduction of the capillaries in the SCP; **(B)** Reduction of the capillaries in the DCP; **(C)** Retinal thinning of the inner retinal layers (blue arrow). SCP, superficial capillary plexuses; DCP, deep capillary plexuses.

Typical SCM (tSCM) was defined as the association of the areas of thinning of the extrafoveal retina and the reduction and loss of vascularization in both the SCP and DCP (Figure 1). We regarded the enlargement of the FAZ as tSCM. In atypical SCM (aSCM), there is no relationship between the thinning of the macula and the perfusion in both the SCP and DCP. Thus, aSCM is defined as the thinning of the extrafoveal retina without any reduction of the perfusion in both the SCP and SCD or a reduction of perfusion on both plexuses without any reduction of retinal thickness. In this study, we considered two groups for statistical analysis, patients without sickle cell disease maculopathy (SCDM) and those with SCDM, which included tSCM and aSCM separately.

### Statistical analyses

Statistical analyses were performed using SPSS (IBM SPSS Statistics, version 28.0). The normal distribution of quantitative data was tested by performing the Shapiro–Wilk test. When normal distribution was obtained once, quantitative data were expressed as means, and a comparison of means was performed by conducting an unpaired Student's *t*-test or *Z*-test, according to the sample size. Qualitative variables were expressed as effectives and percentages and analyzed by performing a Khi-squared test. When the independence of the measures was jeopardized, especially when testing the two eyes of the same subject, we performed generalized estimating equations and intraclass correlation. *P* < 0.05 were considered statistically significant.

## Results

Two hundred and sixty-four eyes of 132 consecutive SCD patients were included in this study. The quality of the OCT-A of one eye of an SS patient was too poor to be analyzed, so the data of this eye were not included in the study. The data were then calculated on 263 eyes. Of the 132 SCD patients, 105 (50 female patients, 47.6 %) had an SS genotype and 27 (16 female patients, 59.2 %) had an SC genotype. The demographic characteristics of these patients are summarized in Table 1. Mean age was 29.7 +/−10.4 years and 35.6 +/−12.4 years in SS and SC patients, respectively, with a significant difference between the two groups (p < 0.01). The mean LogMar best-corrected visual acuities (BCVA) were not statistically different between the two genotype groups (*p* = 0.08). The lowest BCVA was 0.4 LorMar in both eyes of a patient belonging to the SS group. The visual acuity of patients in both groups and the stages of peripheral retinopathy according to Goldberg's classification are summarized in Table 1. The difference of repartition of the SS or SC patients according to Goldberg's classification was statistically significant (*p* < 0.001).

**Table 1:** Demographic characteristic of patients with SS sickle cell disease or SC sickle cell disease.

		**All patients**	**SS patients**	**SC patients**
Number of patients		132	105	27
Sex (female) *n* (%)		66 (50.0 %)	50 (47.6 %)	16 (59.2 %)
Mean age (SD)		30.9 (10.1)	29.7 (10.4)	35.6 (12.4); SS/SC *p* < 0.001
		**Eyes/All patients**	**Eyes/SS patients**	**Eyes/SC patients**
Number of eyes		263	209	54
Mean best-corrected visual acuity (SD)		0.02 (0.19)	0.02 (0.21)	0.01 (0.02); SS/SC *p* = 0.08
Sickle cell disease maculopathy (SCDM)		127	117	10
FAZ		10	8	2
Typical SCDM		92	84	8
Atypical SCDM (paramacular thinning with normal SCP and DCP or reduction of SCP and DCP with a normal macula)		25	25	0
**Peripheral retinopathy**				
Number of eyes (and percentage) according to the stages of Goldberg's classification	0	115 (43.7 %)	104 (49.8 %)	11 (20.4 %)
	1	82 (31.2 %)	62 (29.7 %)	20 (33.4 %)
	2	14 (5.3 %)	12 (5.7 %)	2 (3.7 %)
	3	50 (19.0 %)	29 (13.4 %)	21 (38.9 %)
	4	2 (0.8 %)	2 (4.3 %)	0 (0.0 %)
	5	0 (0.0 %)	0 (0.0 %)	0 (0.0 %)

Best-corrected visual acuity and distribution of the eyes according to Goldberg's classification for all SCD patients, patients with SS sickle cell disease, and patients with SC sickle cell disease.

One SS patient had both FAZ enlargement and typical SCM.

SS patients, patient with SS sickle cell disease; SC patients, patients with SC sickle cell disease; SD, standard deviation; SCP, superficial capillary plexuses; DCP, deep capillary plexuses.

**Figure 2:**
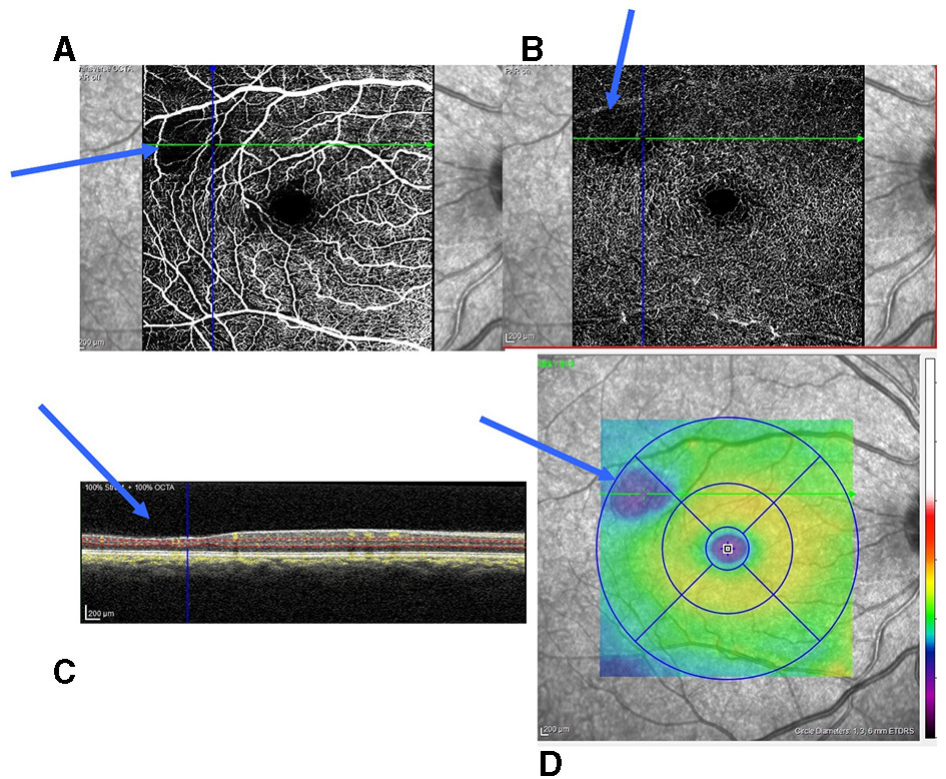
Small parafoveolar retinal thinning and reduction of capillaries in the SCP and DCP. **(A)** Reduction of capillaries in the SCP; **(B)** Reduction of capillaries in the DCP; **(C)** Retinal thinning of the inner retinal layers (blue arrow); and **(D)** Inferotemporal retinal thinning (blue area on the retinal thickness map). SCP, superficial capillary plexuses; DCP, deep capillary plexuses.

In the entire series, SCM was diagnosed in 127 eyes (48.3 %) according to the data from SD-OCT and OCT-A (Figure 2). It was present in 117 (55.9 %) SS patients and 10 (18.5 %) SC patients, with a significant difference (*p* < 0.001). Among SCD patients, typical SCDM was observed in 92 eyes (35.0 %). The enlargement of FAZ could be associated with tSCM. It was observed in 10 eyes (6 patients) in the entire series and was bilateral in four patients. One patient had both typical maculopathy and FAZ enlargement. We observed aSCM in 25 eyes among 17 patients.

In order to be able to use the two eyes of the patients and to analyze them by carrying out generalized estimating equations, we checked whether they were comparable by intraclass correlation. The results confirmed that their data were similar since the intraclass correlation coefficient was over 0.750 for all measurements (Supplementary Table 1).

We observed a statistically significant difference between the presence or absence of SCDM and the genotype. The SCDM is more frequent in SS patients compare to SC patients (*p* < 0.001) (Table 1).

Cerebral vasculopathy was present in 28 patients (26 SS patients and 2 SC patients). We did not have the MRI for 12 patients. The repartition of eyes according to Goldberg's PSR classification and the presence of either eventual SCDM or cerebral vasculopathy are summarized in Table 2. There was a statistically significant relationship between the presence or absence of SCDM and peripheral sickle cell retinopathy (p < 0.001). There was also a statistically significant relationship between the presence of vasculopathy and the stage of PSR according to Goldberg's classification of the eyes of our patients with SCD, as determined by generalized estimating equations (*p* < 0.001) (Table 2).

**Table 2:** Distribution of eyes according to the stages of Goldberg's classification, the presence or absence of macular thinning, and the presence or absence of cerebral vasculopathy.

		**Macula**		**Generalized estimating equations; *P*-value**	**Cerebral vasculopathy**		**Generalized estimating equations; *P*-value**
		**Normal macular thickness**	**SCDM**		**No**	**Yes**	
Number of eyes according to the stages of Goldberg's classification for each group	Stage	137	126		207	56	
	0	56	59	**< 0.001**	40	29	**< 0.001**
	1	44	38		68	13	
	2	8	6		11	2	
	3	28	22		40	10	
	4	1	1		0	2	
	5	0	0		0	0	

The bold values indicate the statistically significant results.

There was a significative statistical difference in the repartition of cerebral vasculopathy according to the genotype (*p* < 0.001) (Table 3A). We observed a significative statistical relationship between the presence of cerebral vasculopathy in our patients and the observation of SCDM in at least one eye (*p* 0.049) (Table 3B).

**Table 3A:** Repartition of eyes according to the presence or absence of cerebral vasculopathy in patients with or without sickle cell maculopathy (SCDM) and statistical analysis of this repartition (using GEE).

		**Macula**		**Generalized estimating equations; *P*-value**
		**Normal macular thickness**	**SCDM**	
Number of patients		119	121	
Cerebral vasculopathy	Yes	20	39	**0.049**
	No	99	85	

SS patients, patient with SS sickle cell disease; SC patients, patients with SC sickle cell disease. SCDM ≥ patients. The bold values indicate the statistically significant results.

**Table 3B:** Repartition of eyes according to the presence or absence of cerebral vasculopathy in patients with SS sickle cell disease or SC sickle cell disease and statistical analysis of this repartition (Khi^2^).

		**SS**	**SC**	**Khi^2^; *P*-value**
Number of patients		96	27	
Cerebral vasculopathy	Yes	26	2	**< 0.001**
	No	70	25	

SS patients, patient with SS sickle cell disease; SC patients, patients with SC sickle cell disease. The bold values indicate the statistically significant results.

We found a statistically significant difference between SC and SS patients in all biological parameters (Table 4A). These data were consistent with possible anemia in SS patients, as well as hemolysis, with a significant elevation of the bilirubin and LDH in the SS group. However, multivariate analyses did not show any differences between the types of SCDM or the risk of vasculopathy according to biological parameters (Table 4B). A significant relationship was present for biological factors of anemia but not for hemolysis and the stage of peripheral sickle cell retinopathy.

**Table 4A:** Repartition of eyes according to the biological values of patients with SS sickle cell disease or SC sickle cell disease.

	**Normal range**	**SS**	**SC**	***P*-value multivariate test**	***P*-value intersubject analysis**	**Cerebral vasculopathy**				***P*-value multivariate test**
		**Value (SD)**	**Value (SD)**			**No**		**Yes**		
						**Value (SD)**	**N eyes**	**Value (SD)**	**N eyes**	
Hb	13–16 g/L	9.0 (1.8)	11.7 (1.6)	**< 0.001**	**0.015**	9.4 (2.2)	69	9.4 (1.6)	24	0.61
HbS	0 %	75.0 (17.2)	49.8 (1.4)		**< 0.001**	72.6 (16.3)		72.7 (21.0)		
Reticulocyte count	20–80 10^9^/L	230.6 (136.3)	132.0 (43.4)		**0.021**	209.7 (112.5)		249.0 (194.3)		
MCV	78–98 fL	88.7 (12.9)	71.3 (7.2)		**< 0.001**	85.0 (13.8)		90.2 (15.0)		
Bilirubine	< 21 mol/L	48.7 (38.0)	25.0 (16.9)		**0.015**	44.5 (36.3)		51.2 (41.8)		
LDH	< 248 UI	411.3 (140.8)	294.8 (77.9)		**0.009**	401.1 (145.7)		400.7 (115.3)		

SS patients, patients with SS sickle cell disease; SC patients, patients with SC sickle cell disease; Hb, hemoglobin; HbS, hemoglobin S level; MCV, mean corpuscular volume; AST, level of aspartate aminotransferase; LDH, level of *lactate dehydrogenase*. The bold values indicate the statistically significant results.

**Table 4B:** Repartition of eyes according to biological values, the presence or absence of macular thinning, and the presence or absence of cerebral vasculopathy.

	**Normal range**	**Macula**				***P*-value; generalized estimating equations**
		**Normal macular thickness**		**SCDM**		
		**Value (SD)**	**N eyes**	**Value (SD)**	**N eyes**	
Hb	13–16 g/L	9.8 (2.2)	39	10.21 (2.13)	56	**0.034**
HbS	0 %	68.7 (20.0)		65.92 (17.76)		0.12
Reticulocyte	20–80 10^9^/L	206.3 (132.8)		211.04 (158.01)		0.28
MCV	78–98 fL	85.4 (13.1)		83.24 (13.27)		**0.003**
Bilirubin	< 21 μmol/L	40.5 (31.5)		35.94 (33.99)		0.61
LDH	< 248 UI	383.6 (147.9)		366.57 (139.95)		0.54

SS patients, patients with SS sickle cell disease; SC patients, patients with SC sickle cell disease; Hb, hemoglobin; HbS, hemoglobin S level; MCV, mean corpuscular volume; AST, level of aspartate aminotransferase; LDH, level of lactate dehydrogenase. The bold values indicate the statistically significant results.

In the multivariate analysis of biological factors of anemia or hemolysis, we observed a significant relationship between reticulocytes and bilirubin, as well as the existence of SCDM and cerebral vasculopathy (*p* < 0.001 and *p* = 0.013, respectively) (Table 4C).

**Table 4C:** Multivariate test for sickle cell maculopathy and cerebral vasculopathy.

	**SCDM/cerebral vasculopathy**	
	**Multivariate test**	***P*-value intersubject analysis**
Hb	**0.025**	0.51
HbS		0.58
Reticulocyte		**< 0.001**
VGM		0.72
Bilirubine		**0.01**
LDH		0.43

SCDM, sickle cell disease maculopathy; SS patients, patient with SS sickle cell disease; SC patients, patients with SC sickle cell disease; Hb, hemoglobin; HbS, hemoglobin S level; MCV, mean corpuscular volume; AST, level of aspartate aminotransferase; LDH, level of *lactate dehydrogenase*. The bold values indicate the statistically significant results.

## Discussion

Although the physio pathogeny of peripheral sickle cell retinal damage is beginning to be well understood, the mechanisms leading to SCM are not clearly defined. However, it is important to identify the risks factors, notably blood parameters, which could be involved in their occurrence. The knowledge of such risks factors could help modify the treatment of SCD patients in order to prevent retinal complications and, perhaps, other systemic complications, especially cerebral vasculopathy (12).

Since retinal internal layers share the same histological nature as the central nervous system, some authors have hypothesized that thinning of the macula shares the same mechanism as cerebral vasculopathy in SCD patients (6). In SCD patients, cerebral vasculopathy was found to result in progressive global gray and white matter atrophy (13, 14). Its mechanisms include nitric oxide deficiency and/or increased expression of VCAM-1 and other adhesion molecules (1). In addition, such cerebral complications may occur early in life, often in children (15). Some studies have indicated that SCM can also appear in children and reported it in this population (10, 16, 17). Martin reported that the frequency of SCM in children is almost the same as in adults in series, leading to suspicions regarding early occurrence in most patients (18). However, little is known about SCM's later evolution. No longitudinal studies have investigated the increase in ischemic areas over time, particularly in young adults. In the ophthalmological subset of an observational study conducted by Martin, data obtained on red blood cells (RBC) deformability led the researcher to suspect that SCDM could be a marker of cerebral oxygenation (18).

In our large series, we observed a difference of prevalence of vasculopathy according to the presence or absence of typical or atypical SCM and a positive correlation according to these parameters. Thus, our results seem to confirm a direct link between the occurrence of SCM and vasculopathy, although our study is focused on young adults. SCM seems to occur mainly in children, as do most silent strokes. Furthermore, Martin could not confirm whether SCM predicts cerebral vasculopathy in children (18). He only found arguments for a similar mechanism in both complications. Our data should also be regarded as complementary arguments for the existence of a similar mechanism for the occurrence of SCDM and cerebral vasculopathy.

We did not study the severity of SCDM. In our series, patients did not have poor vision due to this macular involvement. Thus, we cannot confirm Lim's affirmation that a past history of stroke can be considered a risk factor for more severe SCM in adults (19). However, we found a statistical difference in the prevalence of cerebral vasculopathy according to PSR stages. Cerebral vasculopathy was more prevalent with an increase in the of severity of the stages of PSR. This is in accordance with previous studies that reported an association between the prevalence and/or severity of vasculopathy according to PSR stages (12, 17, 19–21). In addition, there was a statistical difference in SCDM according to the PSR stages in our series. Other authors did not confirm such an association. Bachmeier considered that SCM occurs irrespective of PSR in a study of German patients (22). Only Lim reported that macular thinning increased with PSR stages (19).

However, Lim and Sahak reported a higher frequency of SCDM in SC patients (19, 20, 23). Contrarily, we observed a higher frequency of SCDM in SS patients, as did Fares and Ong (12, 16). This could have been due to the small number of SC patients in our series. It could be considered that the higher number of SS patients masked the relationship with PSR stages, which is more frequently observed in SC patients. However, Hussnain suggested another mechanism. He considered anemia a major risk factor (23). Such a correlation was also reported in other studies (12, 17, 18). SS patients in our series had more severe regenerative anemia than SC patients. They also presented evidence in support of hemolysis, as indicated by an increase in bilirubin and LDH. These results are in accordance with previous data, especially those of Hussnain, as well as those of Martin who considered SCDM a marker of cerebral oxygenation, as previously noted (18). In addition, some patients with SCDM had a severe reduction or disappearance of retinal vascularization in both the superficial and deep plexuses without thinning of the retina. This might correspond to an early stage of SCDM. Ong described similar findings in two patients with paracentral acute middle maculopathy, where OCT-A revealed a disappearance of parafoveal vascularization (24). A similar chronic mechanism could be suspected with anemia inducing a reduction in vascularization in the temporal region of the macula, which corresponds to a watershed at the horizontal raphe, an area of terminal vascularization. Thus, the pathophysiological mechanisms of macular involvement seem to be related to local ischemia. This is not in contradiction with the association between ischemia or local ischemia and brain damage, which may also share the same mechanism in children.

We did not show any relationship between SCDM and biological data or between cerebral vasculopathy and biological data. It must be pointed out that there were only signs of regenerative anemia with elevated reticulocytes in the groups of patients with SCDM and cerebral vasculopathy, as well as an increase in bilirubin in the latter group of patients. However, in the multivariate analysis, a statistical correlation between reticulocytes and bilirubin and SCDM and cerebral vasculopathy considered together was observed. Along with the signs of anemia already mentioned, Fares considered that biological markers of hemolysis could be associated with the risk factors of SCDM (12). We then considered that hemolysis could be a risk factor for cerebral vasculopathy since it could increase the risk of anemia.

Our study has several limitations. As noted, the biological data were collected during follow-up visits on young adults. Thus, we do not have data regarding when the macular abnormalities occurred. In addition, patients might have experienced hemolysis crises a few days before the follow-up, and they could still have had sequelae from these episodes, which are usually more frequent in SS patients. Nevertheless, the concordance of the values found in the different groups tends to prove that biological abnormalities could be a structural state. We did not measure the vascular “flow” at the different incriminated areas. These measurements depend on the diameter of the vessels, which is not always precisely known. Thus, we only considered vascular loss on OCT-A, confirmed by experienced observers, in both the superficial and deep levels corresponding to two different plexuses. The size of our group of SC patients was relatively small, which could explain why some anomalies were absent or did not reach significance in this genotypic group. Finally, this study was retrospective and limited to a single visit. It would be interesting to perform a longitudinal evaluation of purely retinal or purely vascular lesions to specify their evolution.

## Conclusion

This study obtained several important findings. Firstly, our results showed a relationship between SCD cerebral vasculopathy and SCDM since there was a significant association between the presence of these two complications. In addition, there was a relationship between these two complications and anemia or local ischemia. Thus, since retinal and brain tissues share some characteristics, it seems that these complications can share the same mechanism as well. Secondly, our biological data tended to prove that there was relationship between hemolysis markers and cerebral vasculopathy and SCDM. This could be an argument for preventive treatments carried out on these patients to avoid vaso-occlusive crises.

## Data availability statement

The raw data supporting the conclusions of this article will be made available by the authors, without undue reservation.

## Ethics statement

The studies involving humans were approved by Local Ethic Committee (CERAPHP Center, Comité d'éthique de la recherche AP-HP Center). The studies were conducted in accordance with the local legislation and institutional requirements. According to the national legislation, patients had not to give a consent, but they were advised that their data could be used anonymously and that they could oppose it.

## Author contributions

CO performed the ophthalmological examination and wrote the main manuscript. EF and AM examined the patients. BR and JA wrote the hematological part of the main manuscript. All authors reviewed the manuscript.

## Supplementary material

Supplementary Table 1 Interclass correlation coefficient, significativity, and Cornbach'alpha for visual acuity, SCDM, and stages of Goldberg's classification data from the right and left eyes.
