# Supplementary material for: Relationship between paramacular thinning, cerebral vasculopathy, and hematological risk factors in sickle cell disease
Source: Front Med (Lausanne). 2023 Aug 28;10:1226210. doi: 10.3389/fmed.2023.1226210 (PMC10493280; doi:10.3389/fmed.2023.1226210)
Supplement: Supplementary Table 1 — Interclass correlation coefficient, significativity, and Cornbach'alpha for visual acuity, SCDM, and stages of Goldberg's classification data from the right and left eyes. [file Table_1.pdf]

	Visual acuity	SCDM	Stage of Goldberg classification
Intraclass correlation coefficient	0.788	0.762	0.863
p (Fisher test)	< 0.001	< 0.001	< 0.001
Cronabch Alpha	0.788	0.762	0.863

Table suppl n° 1
